# Microstructure, Microhardness, and Wear of a Rapidly Solidified Al–20Sn–1Cu Alloy with Bi Addition

**DOI:** 10.1021/acsomega.5c12710

**Published:** 2026-03-12

**Authors:** Vinicius Leme Andrade, Sarah De Albuquerque, Rodrigo André Valenzuela Reyes, José Eduardo Spinelli

**Affiliations:** Department of Materials Science and Engineering, 541490 Federal University of São Carlos, Rod. Washington Luis, São Carlos, SP CEP 13565−905, Brazil

## Abstract

This study investigates the effects of Bi addition on the microstructure, hardness, and wear behavior of a rapidly solidified Al–20Sn–1Cu alloy, aiming to clarify Bi’s role under cooling rates comparable to those experienced during twin-roll casting (TRC). Alloys with and without 2 wt % Bi were solidified in a stepped graphite mold, under cooling rates between 260 and 409 °C/s. Microstructural analyses revealed that Bi did not alter the primary cellular/dendritic growth of the α-Al matrix but significantly modified the interdendritic Sn-rich phase, promoting its fragmentation. CALPHAD predictions confirmed the occurrence of liquid phase separation in the Bi-containing alloy, consistent with the formation of finely dispersed Bi-rich regions observed experimentally. Microhardness increased with Bi addition and with increasing cooling rate. Wear ball-cratering tests showed that Bi addition markedly reduced the worn volume, particularly for rapidly solidified samples, and led to a distinctive evolution of the dimensional wear coefficient (k). This improvement is attributed to the combined effects of microstructural refinement and increased hardness. The worn surfaces exhibited predominantly adhesive wear, characterized by material transfer from the steel counterface and minor oxidation. Overall, the results demonstrate that Bi addition is an effective strategy to restore or enhance the tribological performance of Al–Sn alloys produced under rapid solidification conditions relevant to the TRC process.

## Graphical abstract

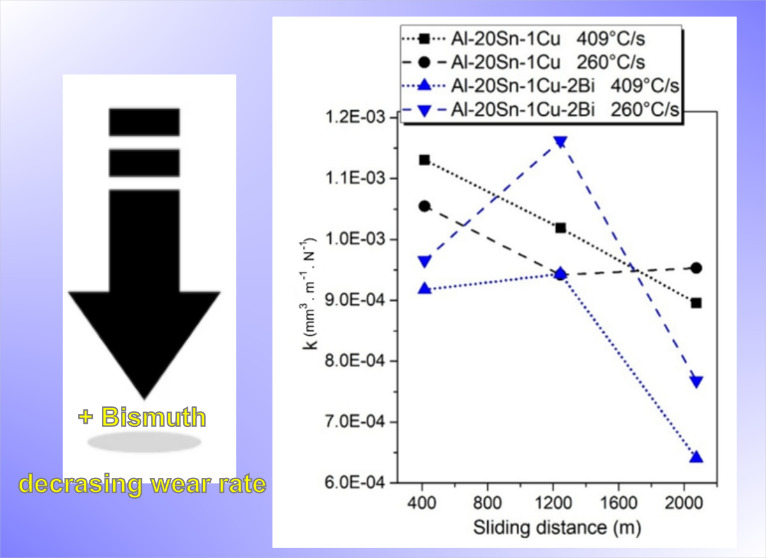

## 1 Introduction

Focus on the impact of bismuth (Bi) on the microstructure and mechanical properties of Al–Sn alloys has emerged as an area of interest due to its implications for developing lightweight, wear-resistant alloys for automotive and aerospace applications.^1–3^ Al–Sn alloys have been valued for their self-lubricating properties, with early studies focusing on microstructural control through alloying and processing techniques.^4,5^ Recent literature demonstrated that Bi is a soft phase that can not only modify eutectic structures but also enhance machinability and wear resistance^6,7^ The relevance of this research relies on the need for alloys that reduce frictional losses, which account for approximately 23% of global energy use.^2,8^ This contributes to energy efficiency and sustainable industrial innovation in line with the United Nation Sustainable Development Goals.^9^ Moreover, the practical relevance of Al–Sn alloys lies in the demand for combining high performance with cost-effective manufacturing, for which twin-roll casting (TRC) provides an energy- and time-efficient near-net-shape route,^10^ becoming largely used for processing self-lubricating alloys. Although the role of Bi in the Al–Sn based alloys is relatively well-known as already mentioned, its effects under high solidification rates, as typically obtained through TRC, remain unclear, warranting further investigation into its influence on microstructure, wear and mechanical behavior.

Several studies have reported the capabilities of Bi concerning both grain-refining and eutectic-modifying, while few discrepancies exist regarding optimal Bi concentrations and their impact on hardness, tensile strength, and wear.^11,12^ Contrasting findings also emerge related on Bi interaction with other alloying elements such as Mg and Cu, which can form intermetallic phases like Mg_3_Bi_2_, potentially reducing machinability.^13,14^ This knowledge gap limits the ability to tailor Al–Sn–Bi alloys for specific engineering applications, risking suboptimal performance and premature failure. Moreover, very few reports related to TRC have been found, with focus on alloys other than Al–Sn. The TRC process significantly influences the wear behavior of Al–Sn alloys by enhancing their microstructural properties and mechanical performance. Besides as-cast microstructure impact, the TRC technique also promotes dynamic recrystallization and grain rotation, which contributes to improved wear resistance. This is particularly evident in the wear volume reduction observed under varying loads, where higher substructure and deformation textures lead to less wear.^15^ Additionally, the control of surface quality through the generation of an Al coating layer during casting enhances the interfacial heat transfer, resulting in better solidification output and reduced surface defects, which can also affect wear characteristics.^16^

In the present investigation, two alloys were produced: a reference Al–20Sn–1Cu (wt %) and a Bi-modified Al–20Sn–1Cu–2Bi (wt %), both rapidly solidified using a graphite stepped mold with reduced melt thicknesses ranging from 1.5 to 3 mm. Both alloys were evaluated by wear testing and Vickers microhardness measurements. The resulting data correlated with the measured solidification rates, which reached maximum values slightly above 400 K/s, similar to those experienced during TRC. The purpose of this method and study is to emulate real solidification cooling rates experienced in TRC conditions and obtain well-controlled samples to correlated investigation of microstructure, hardness, and wear behavior, thereby enabling the assessment of Bi effects and solidification kinetics under conditions close to those found in the TRC industrial processes.

## 2 Materials and Methods

The base alloy (Al–20Sn–1Cu) selected for this study corresponds to a commercial Al–Sn alloy commonly used in bearing applications, SAE 783.^17^ Bismuth was added to promote the formation of a Bi-rich phase. To support this design, the solidification path of the investigated alloys was calculated from the equilibrium phase diagram using the CALPHAD method with Thermo-Calc software and the TCAL 7.1 database.

The Al–20Sn–1Cu and Al–20Sn–1Cu–2Bi alloys were produced in an induction furnace (Inductotherm VIP Power-Trak, Rancocas, NJ, USA) by melting commercially pure elements in their stoichiometric proportions. Unless otherwise indicated, all compositions are expressed in weight percent.

To simulate the rapid solidification conditions typically observed in industrial TRC, a graphite stepped mold was employed as a laboratorial scale alternative processing route. It should be emphasized that, while the stepped graphite mold enables cooling rates comparable to those reported for the TRC, the present experimental approach does not reproduce the shear deformation, strain accumulation, or dynamic recrystallization effects inherent to the TRC process. Therefore, the comparison here is intentionally limited to cooling-rate-controlled solidification phenomena, which primarily govern cellular/dendritic scale refinement.

This approach aimed to establish a single, standardized method for sample fabrication, enabling a direct comparison between the alloys with and without Bi. Moreover, each mold wall thickness corresponds to a distinct solidification condition, which is more severe (i.e., higher cooling rates) for thinner cast sections and less severe for thicker ones. As can be observed in Figure 1, the thickness of the sections varied from 1.0 mm to 3.5 mm. Type-K thermocouples were inserted inside each mold section to monitor the temperature evolution during solidification. Data was recorded at 20 Hz. The resulting cooling rates were later used to correlate microstructural coarsening, wear behavior, and microhardness. It is worth mentioning that the solidification of the Al–20Sn–1Cu and Al–20Sn–1Cu–2Bi alloys in a graphite mold was conducted under comparable thermal conditions representative of rapid industrial solidification.

**Figure 1:**
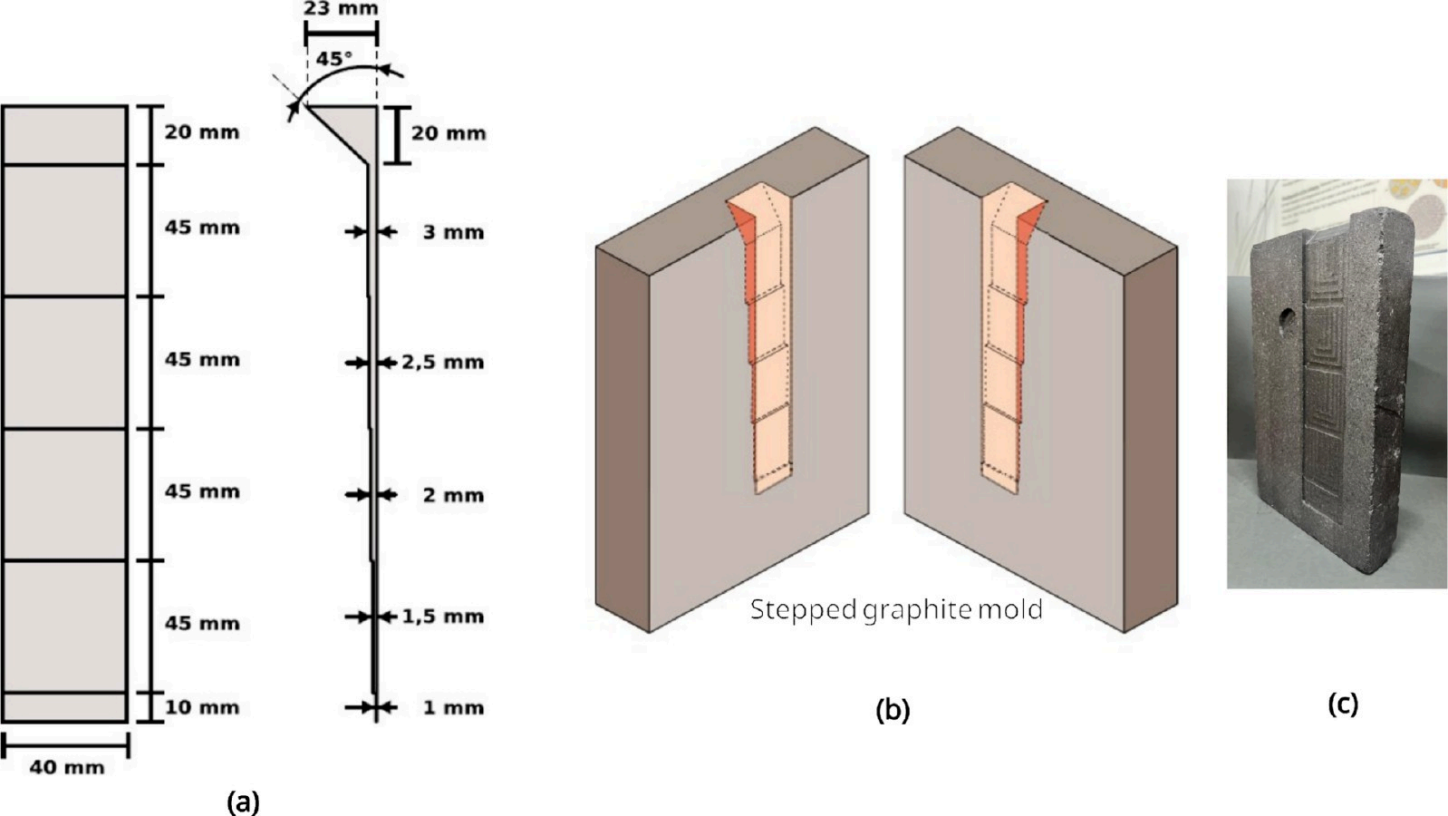
(a) Technical drawing, (b) 3D illustration of the stepped graphite mold, and (c) photograph of the mold (dimensions in mm). The 1 mm-samples were not analyzed due to the presence of cracks.

Metallographic preparation followed conventional grinding, polishing, and chemical etching procedures. The samples were etched by immersion for 12 s in Keller’s reagent (2 mL HF, 3 mL HCl, 5 mL HNO_3_, and 75 mL H_2_O) to reveal the dendritic structure. Optical microscopy (Olympus BX41M-LED, Japan) was employed to examine the general microstructural features, while quantitative analyses were performed using ImageJ software.^18,19^ Detailed microstructural and compositional analyses were performed using a field-emission scanning electron microscopy (FEG-XL30, Philips, Netherlands) equipped with a backscattered electron detector (BSE, Bruker, USA).

The average length scale of the α-Al phase (λ_*A*_) was measured using a procedure adapted from the Heyn linear intercept method.^20^ In this approach, the λ_*A*_ was calculated by determining the ratio of dendritic arms intersected by a line of known length. Image analysis was performed using ImageJ software, where micrographs were automatically segmented into dendritic and interdendritic regions through a machine-learning-based classification algorithm considering color and morphological features.^21^ Additional methodological details can be found in a previous study.^22^

Phase identification was performed by X-ray diffraction (XRD) using a Bruker D8 Advance ECO diffractometer (Massachusetts, USA) with Cu–Kα radiation (λ = 1.5406 Å). Diffraction patterns were acquired in the 2θ range of 10–90° with a step size of 0.02°.

For the wear tests, and microhardness measurements, the thinnest and thickest sections of the cast were selected. This selection aimed to provide a significant contrast between the results and to evaluate the influence of solidification kinetics on the mechanical behavior and wear of both alloys.

The microhardness measurements were obtained using a Vickers microhardness tester (Shimadzu HMV-G 20ST, Japan). Reported values correspond to the average of at least 20 indentations performed with a 0.5 kg load and 10 s dwell time.

Tribological behavior was evaluated by means of a ball-cratering tribometer operating in a fixed-ball mechanical configuration. The tests were performed using a microadhesive ball-cratering wear apparatus,^23,24^ in which a rotating AISI 52100 steel ball (25.4 mm diameter) was rolled against the sample surface under a normal load of 0.6 N at 260 rpm, following the procedure described in ref 25. A schematic representation of the used wear tester is shown in Figure 2.

**Figure 2:**
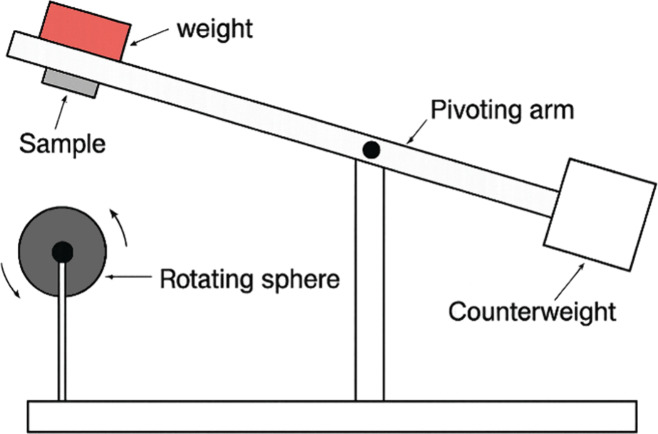
Schematic diagram and details of the sliding wear tester.

The tests produced spherical cap-shaped craters on the sample surfaces. Wear volumes (*V*_w_) were calculated using eq 1, based on the arithmetic mean of four measured crater diameters (*d*) and the radius of the sphere (*R*).^26^

$$V_{w}=\frac{\pi \cdot d^{4}}{64\cdot R}$$ 1

The experiments were performed for durations of 20, 60, and 100 min. To isolate the influence of microstructure, all tests were conducted under dry conditions, without lubricants or abrasive particles.

Gee et al.^27^ demonstrated the reproducibility of the ball-cratering test using standard wear-resistant coatings, reporting a high consistency of wear depth measurements with a standard deviation of less than 5%. Similarly, promising reproducibility results were reported by Macedo et al.,^28^ who observed a standard deviation below 10% for wear volume measurements in microabrasion tests using the ball-cratering device.

Wear behavior was further analyzed using the dimensional wear coefficient (k) in eq 2, derived from the classical Archard relationship. This parameter represents the volume of material removed per unit sliding distance per unit normal load:^29^

$$k=\frac{V_{w}}{m\cdot N}$$ 2

Where *V*_W_ is the worn volume in mm^3^, *m* is the sliding distance in meters, and *N* is the test load in N.

## 3 Results

### 3.1 Equilibrium Solidification Path

CALPHAD calculations performed for both chemistries can be seen in Figure 3. The equilibrium diagrams of mass fraction of phases as a function of temperature reveal that the base Al–20Sn–1Cu alloy exhibits a relatively simple phase evolution dominated by the FCC-A1 (α-Al) phase, with Al_2_Cu and β-Sn (BCT_A5) forming at lower temperatures as a eutectic constituent. In contrast, the Bi-containing alloy (Al–20Sn–1Cu–2Bi, Figure 3b) shows a more complex solidification path, with two distinct liquid regions indicating partial immiscibility. This system also develops an additional rhombohedral phase (RHOMBO_A7) corresponding to solid Bi, which appears at lower temperatures alongside α-Al, β-Sn, and Al_2_Cu. Overall, Bi addition promotes liquid segregation and introduces a soft Bi-rich phase that can act as a solid lubricant.

**Figure 3:**
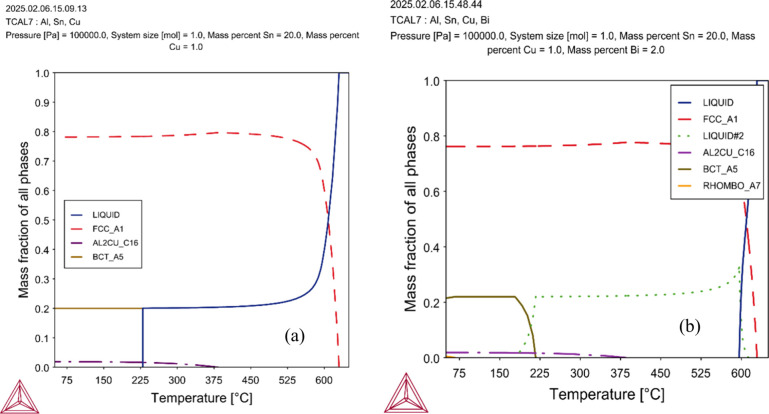
Calculated equilibrium phase fraction diagrams for (a) Al–20Sn–1Cu and (b) Al–20Sn–1Cu–2Bi alloys using the TCAL7 database.

### 3.2 Solidification and Morphology Evolution

The alloys were successfully cast using a stepped graphite mold, which induced different cooling rates based on the section thickness. The thermal analysis from the thermocouples provided the cooling curves and corresponding cooling rates for the different sections,^22,30^ as represented in Figure 4. The cooling rate corresponding to each thermocouple was obtained from the temperature–time data by calculating the derivative of the cooling curve taking as reference the moment when the *liquidus* (630 °C) front intersected the thermocouple. Samples corresponding to the thinnest thickness and therefore the fastest cooling rate (409 °C/s) and thickest thickness and therefore slow cooling rate (260 °C/s) were selected for detailed analysis to represent the microstructural extremes obtained. The micrographs corresponding to these two samples are shown in detail in Figures 5 and 6.

**Figure 4:**
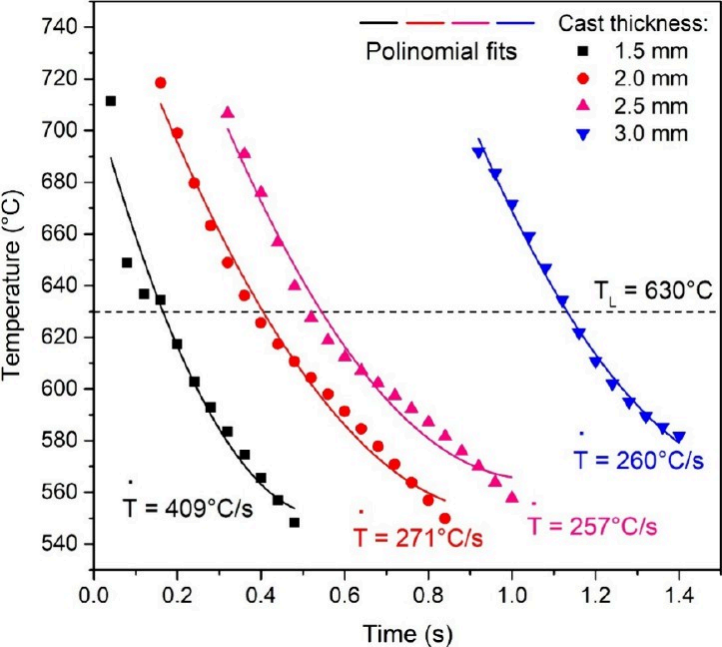
Experimental thermal profiles and respective polynomial fits for the data with the Al–20Sn–1Cu alloy solidified in the stepped graphite mold.

**Figure 5:**
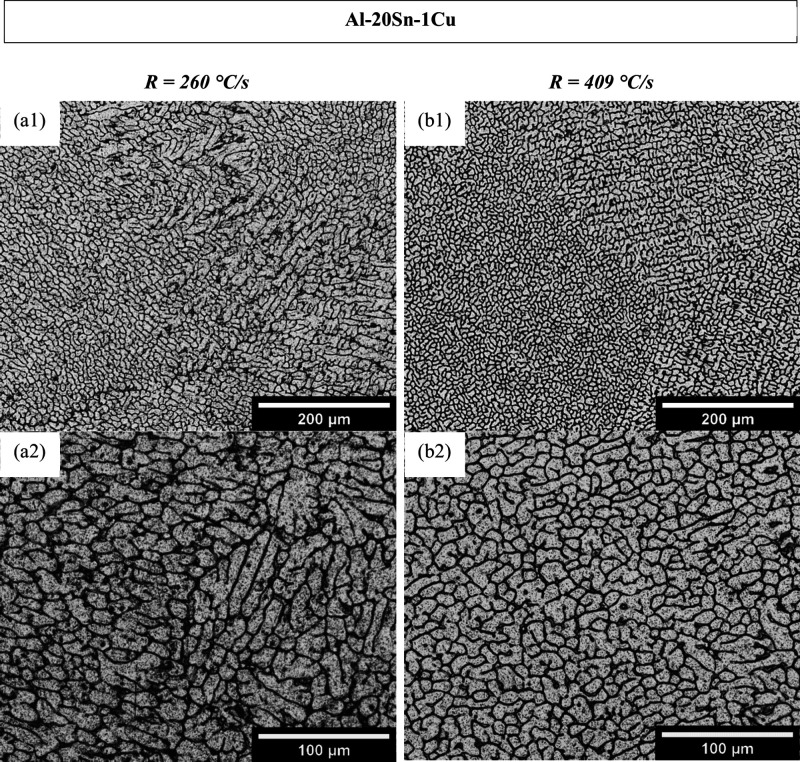
Optical micrographs at different magnifications of the Al–20Sn–1Cu solidified at (a) 409 °C/s and (b) 260 °C/s.

**Figure 6:**
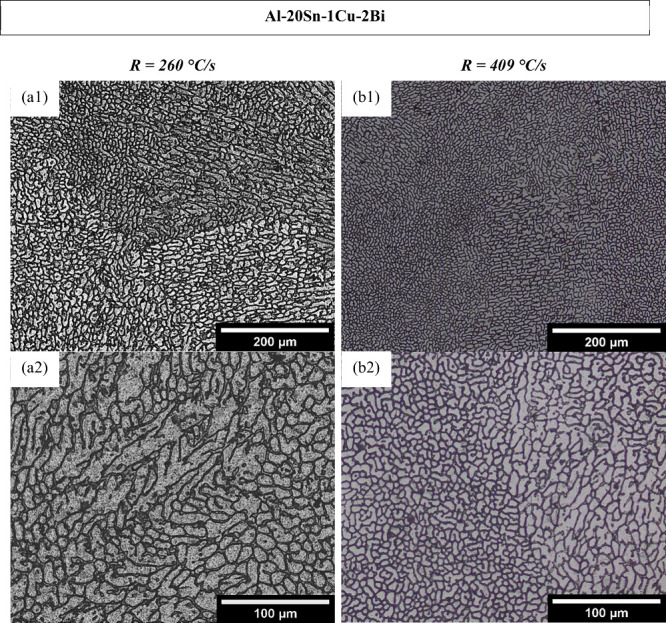
Optical micrographs at different magnifications of the Al–20Sn–1Cu–2Bi solidified at (a) 409 °C/s and (b) 260 °C/s.

Figures 5 and 6 correspond to the microstructures of the Al-20Sn-1Cu and Al-20Sn-1Cu-2 Bi alloys, respectively. In both alloys, the formation of cellular and dendritic patterns is evident. Samples solidified under higher cooling rates exhibit a larger fraction of very refined α-Al cells, whereas coarser cellular and dendritic arrangements are observed in the regions solidified at the lowest cooling rate (260 °C/s, left columns).

The length scale of the α-Al phase (λ_*A*_) was also quantified. For the reference Al-20Sn-1Cu alloy, the average spacing increased from 25.4 to 33.4 μm as the cooling rate decreased. A similar trend was found for the Bi-modified alloy, where (λ_*A*_) increased from 29.7 to 47.7 μm.

When comparing both alloys, the overall cellular and dendritic morphologies remain remarkably similar across the different cooling conditions. The addition of 2 wt % Bi does not significantly alter the primary α-Al growth patterns within the stepped mold, suggesting that Bi did not significantly alter the microstructural evolution.

SEM using a Backscattered Electron (BSE) detector provided compositional contrast, differentiating the phases in both alloys as can be seen in Figure 7a,b. The network-like phase delineating the cell/dendrite boundaries in Figure 7a represents Sn-rich (β-Sn) regions. This contrast arises from the higher atomic number of Sn, which produces stronger backscattered electron signals. The microstructure exhibits a fine and interconnected Sn morphology, uniformly distributed along the α-Al boundaries. A similar distribution of Sn-rich and Bi-containing second phases is observed in the Al-20Sn-1Cu-2 Bi alloy (Figure 7b), however, unlike the ternary alloy, the Bi-containing sample exhibits a noticeably more fragmented interdendritic/cellular network. This feature becomes especially evident in the higher-magnification image of Figure 7c.

**Figure 7:**
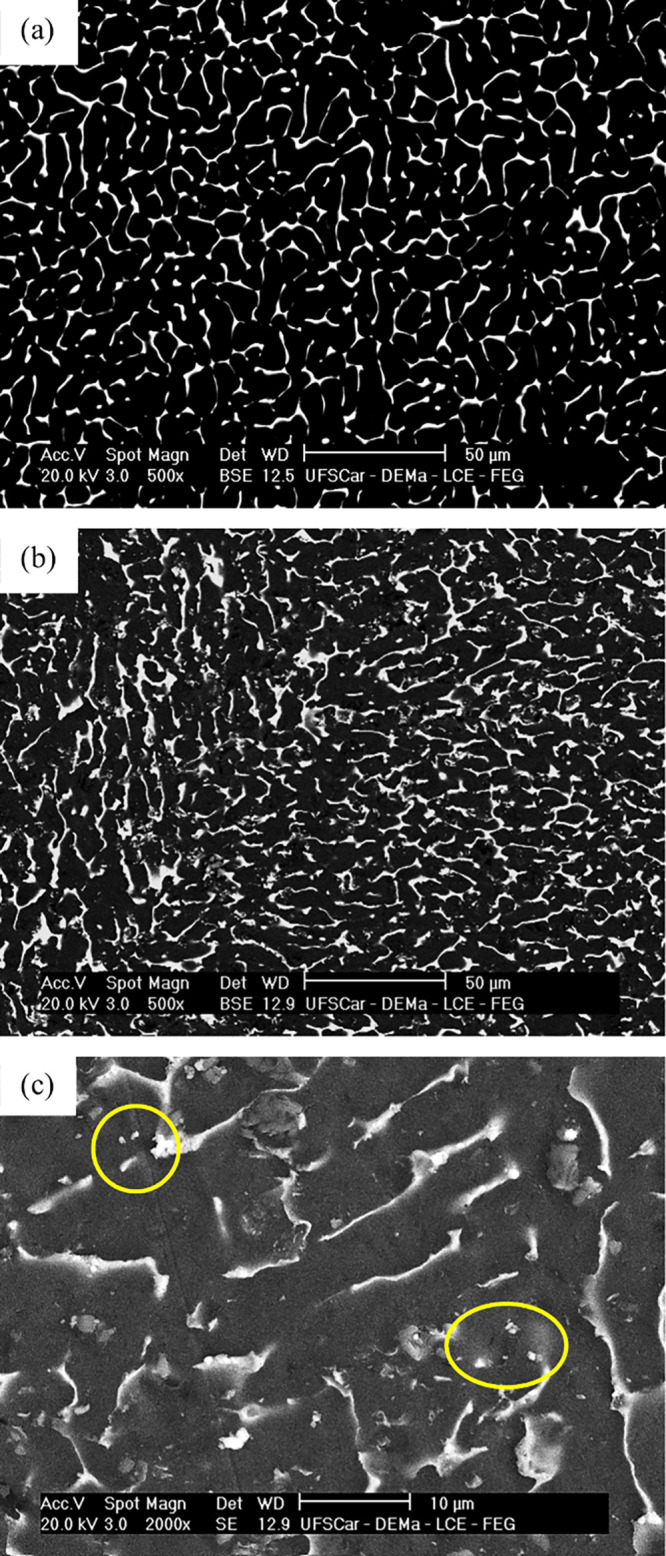
Backscattered electron (BSE) SEM micrographs of (a) Al–20Sn–1Cu and (b) Al–20Sn–1Cu–2Bi alloys solidified at 409 °C/s. (c) High-magnified SEM image of the Al–20Sn–1Cu–2Bi alloy.

### 3.3 Microhardness and Wear Behavior

Vickers microhardness (HV) results can be seen in Figure 8. The samples solidified at a higher cooling rate (409 °C/s) exhibited higher microhardness than those cooled more slowly (260 °C/s). The addition of 2 wt % Bi resulted in increased microhardness compared to the reference alloy for both cooling conditions (260 and 409 °C/s).

**Figure 8:**
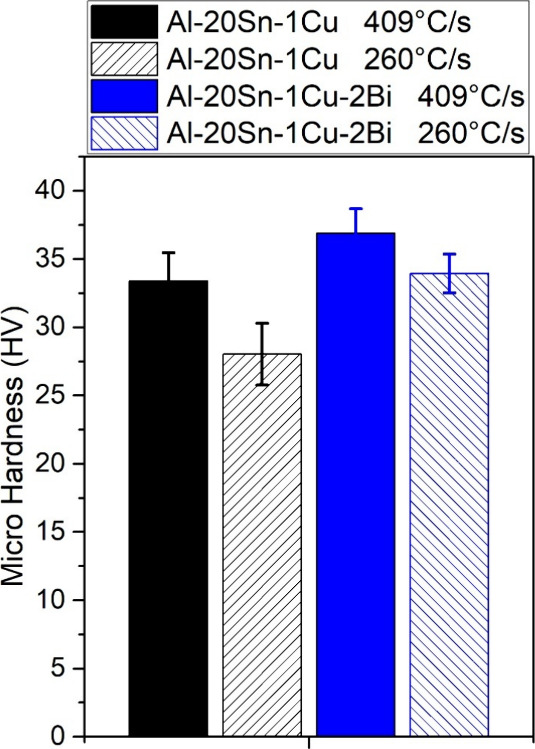
Microhardness (HV) of the Al–20Sn–1Cu and Al–20Sn–1Cu–2Bi alloys solidified at 409 and 260 °C/s.

Although the present study was limited to hardness and wear performance, toughness is also a relevant mechanical property. In this context, the literature indicates that Bi addition can enhance the toughness of Al–Sn alloys at appropriate contents.^1^

The adoption of the eq 1 to calculate the volume of the spherical wear scar was shown to be efficient for wear craters as those typically obtained after the tests (Figure 9). It can be seen that as the test duration increases, the number of rotations or the sliding distance also increases, and as a consequence, worn volume increases, according to Figure 10. For the Bi-free alloy, the influence of cooling rate is modest, with the sample solidified at the lower cooling rate (260 °C/s) exhibiting a slightly higher worn volume, particularly at longer sliding distances. In contrast, the addition of 2 wt % Bi significantly reduces the worn volume, and this improvement is most pronounced at higher cooling rates.

**Figure 9:**
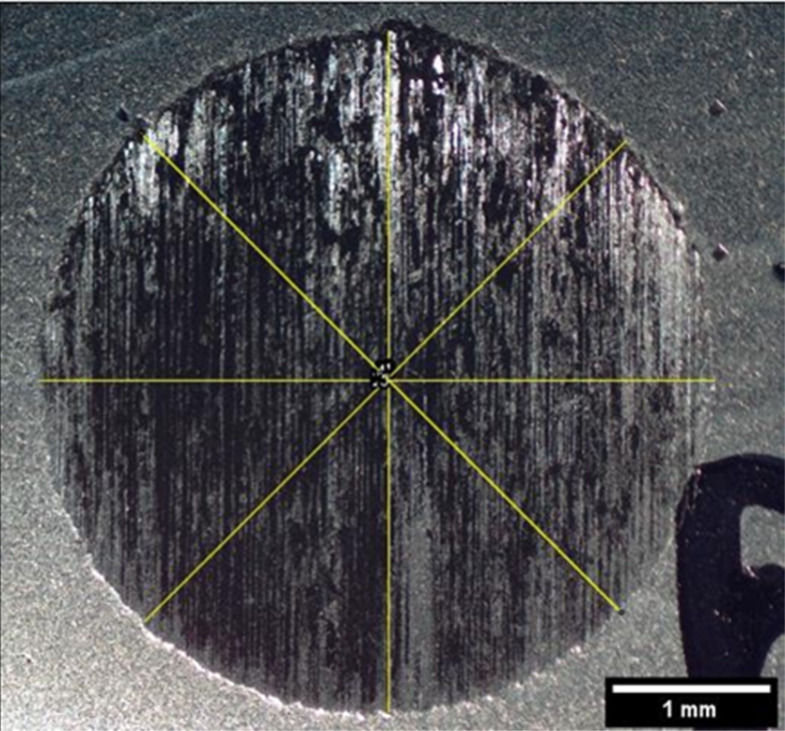
Example of measuring diagonals on a cap generated by wear.

**Figure 10:**
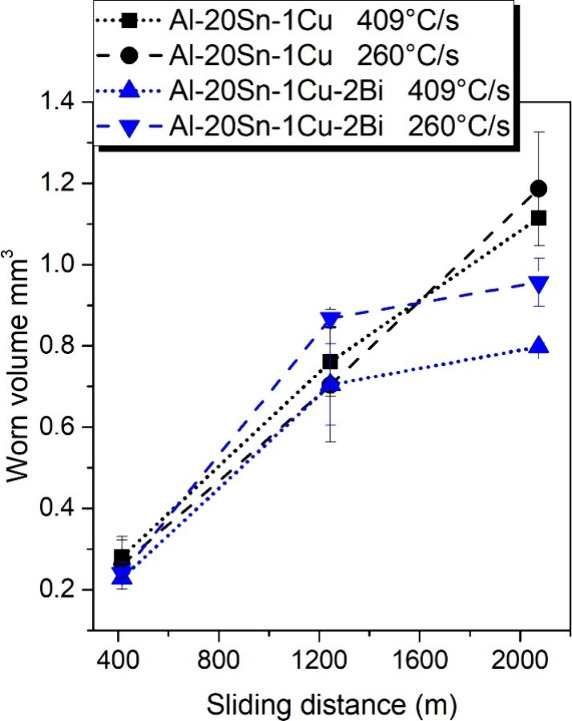
Worn volume as a function of sliding distance for Al–20Sn–1Cu and Al–20Sn–1Cu–2Bi alloys solidified at 409 and 260 °C/s.

## 4 Discussions

### 4.1 Solidification

Thermodynamic calculations using the CALPHAD method (Thermo-Calc + TCAL 7.1) indicate that liquid phase separation in the Al–20Sn–1Cu system occurs only when the Bi content reaches at least 2 wt %. Below this threshold, no immiscibility domain is predicted. With 2 wt % Bi, however, the alloy composition intersects a miscibility gap, leading to spontaneous decomposition of the liquid into two immiscible fractions (*L*_1_ + *L*_2_), one liquid rich in Al and another one rich in Sn/Bi, as indicated by the green dotted-line in the calculated solidification path (Figure 3b). This behavior is driven by the highly positive mixing enthalpy of the Al–Bi and Al–Sn pairs, with the Al–Bi repulsion being particularly strong.^31^

Despite its immiscibility with Al, Bi exhibits high liquid-state miscibility with Sn, forming a single-phase liquid over a broad temperature range. As suggested by Dong et al.,^3^ this has important implications for the solidification path. Part of the Bi-rich liquid can dissolve into the Sn-rich liquid, attenuating the strong tendency of Bi to undergo macrosegregation and upon further cooling, the Bi dissolved in the Sn-rich phase becomes supersaturated and precipitates as a Bi-rich rhombohedral phase. Which implies that even a small Bi addition not only induces liquid-phase separation but also modifies Bi distribution during cooling. The balance between Al–Bi repulsion, Bi–Sn liquid miscibility, and Bi reprecipitation plays a central role in determining the final microstructural arrangement of Bi, which is crucial for optimizing the performance of Al–Sn–Cu–Bi bearing alloys.

One of the primary challenges in manufacturing components from immiscible alloys is controlling the distribution of the secondary phase.^32,33^ In the liquid state, droplets of the minority phase grow mainly through several mechanisms, including diffusion-controlled growth, Brownian motion, Marangoni-driven motion, and Stokes sedimentation,^34^ which typically lead to a heterogeneous dispersion of phases.

In general, the cooling rate has a significant influence on the microstructure of these alloys. Under slow cooling conditions, pronounced spatial phase separation occurs. As the cooling rate increases, the alloy microstructure can be tailored, and the degree of phase separation is largely suppressed.^35^ In the samples analyzed, no evidence of macrosegregation was observed, indicating that the high cooling rates were effective in suppressing the coalescence of the Sn- and Bi-rich phases.

The samples produced using the stepped mold had their cooling rates measured, and the extreme thickness conditions, and consequently the extreme cooling rates, were analyzed with respect to microstructure, microhardness, and wear behavior.

### 4.2 Microstructure

During solidification, the Al-rich matrix develops as the primary phase, while Sn and Bi, both characterized by extremely low solubility in Al, are rejected into the remaining liquid. Consequently, these solute-enriched elements solidify as well-defined networks distributed along the interdendritic and intercellular regions of the α-Al matrix, as shown in Figures 5 and 6.

Oliveira et al.^36^ reported that binary Al–Sn alloys containing more than 10 wt % Sn generally develop fully dendritic microstructures. Their study also demonstrated, however, that cooling rate plays a decisive role in dictating the morphological regime: for a given alloy composition, variations in thermal conditions may promote a transition from dendritic to cellular growth.

The transition at the solid–liquid (S/L) interface during the development of cells and dendrites can be interpreted through the constitutional undercooling criterion for binary alloy solidification. According to this criterion, an increase in solute concentration (C_0_), an increase in growth rate (v), or a decrease in thermal gradient (G) can destabilize the S/L interface, thereby promoting the transition from cellular to dendritic morphologies. However, for a fixed alloy composition, increasing the solidification velocity results in the following sequence of morphological transitions: planar → cellular → dendritic → cellular → planar.^37^ Therefore, the experimental apparatus employed in the present work generated high cooling rates, enabling the formation of high-velocity cells, also referred to as high cooling-rate cells.^36,38^

Incorporating Bi into the alloy did not fundamentally alter this morphological sequence. Both alloys exhibited the same mixture dendritic/cell structure. The results reinforce that cooling rate is the dominant factor controlling α-Al morphological refinement, while Bi addition mainly influences the solute-rich interdendritic structure without disrupting the primary solidification mode.

X-ray Diffraction (XRD) analysis was conducted to identify the crystalline phases present after solidification. The XRD patterns in Figure 11 confirmed the presence of α-Al, and Sn phases in both alloys. The characterization of the θ-Al_2_Cu phase was inconclusive by XRD. This may be attributed to the very low predicted fraction of θ-Al_2_Cu, which is close to the detection limit of the technique, making its identification difficult. As discussed by Cullity and Stock,^39^ the sensitivity for detecting small phase fractions strongly depends on the characterization method, and phases present in very low amounts may remain undetected. The diffractogram for the Al-20Sn-1Cu-2 Bi alloy clearly shows additional peaks corresponding to a crystalline Bi phase.

**Figure 11:**
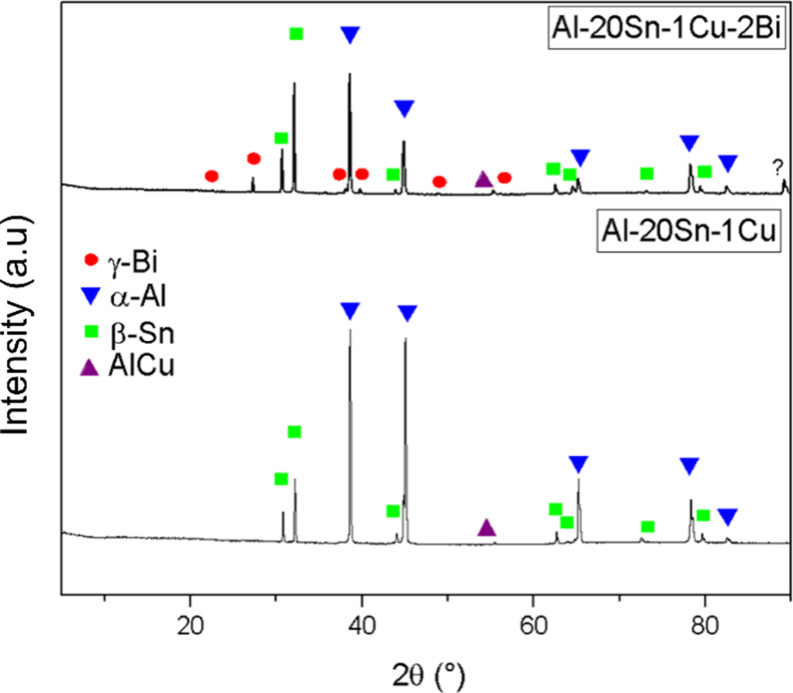
XRD patterns for Al–20Sn–1Cu and Al–20Sn–1Cu–2Bi alloys.

Additionally, energy-Dispersive X-ray Spectroscopy (EDS) dot maps in Figure 12 confirmed the elemental distribution in the Bimodified alloy. The microstructure consists of an Al-rich matrix, as indicated by the continuous distribution of Al. Sn and Bi are concentrated in the interdendritic regions, forming interconnected networks consistent with solute segregation during solidification. Sn shows a pronounced enrichment along these channels, while Bi appears as discrete, finely dispersed pockets within the same regions. In contrast, Cu is uniformly distributed throughout the matrix, indicating full solubility.

**Figure 12:**
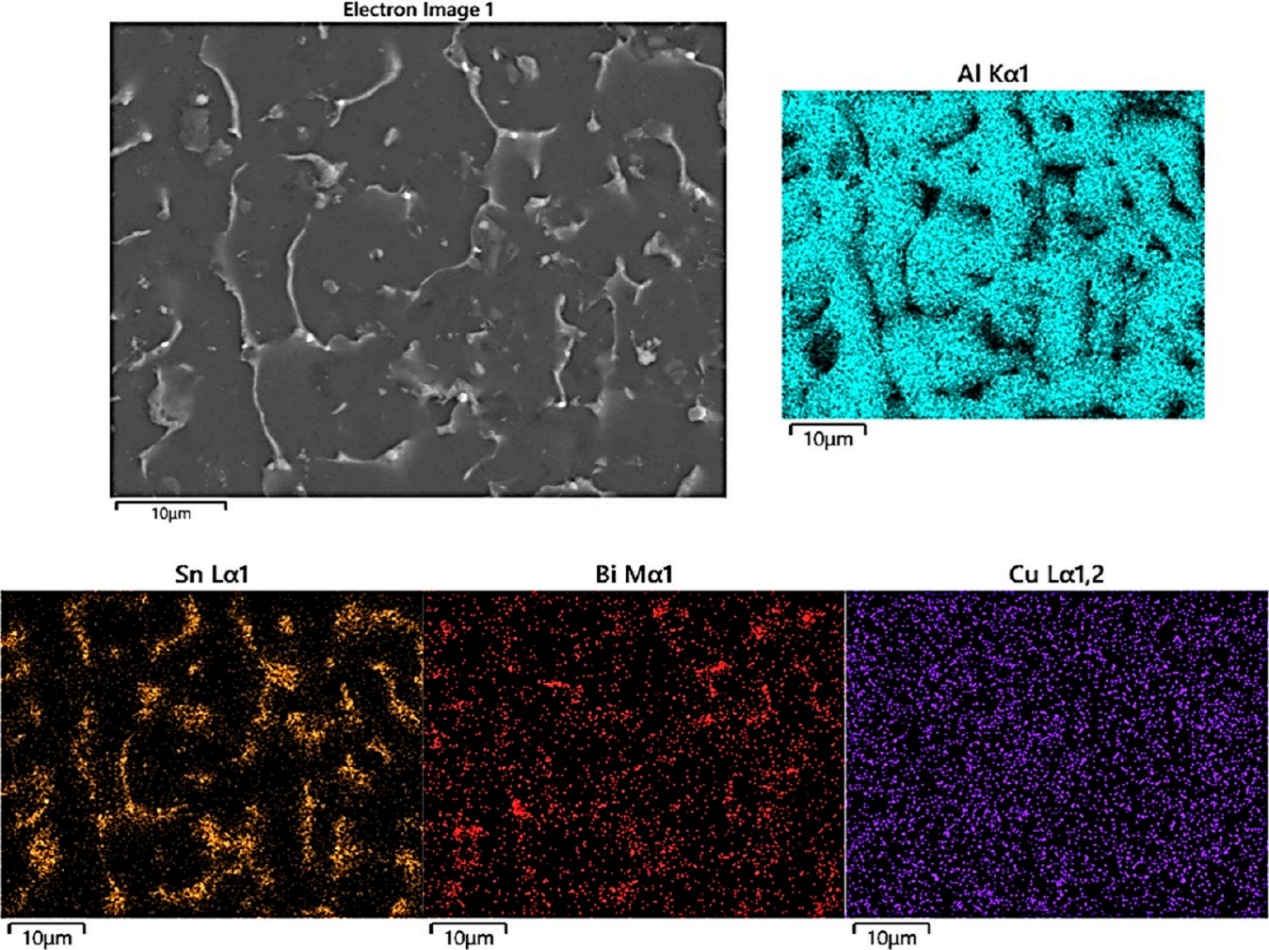
EDS elemental maps of the Al–20Sn–1Cu–2Bi alloy showing Al (cyan) in the matrix, Sn (yellow) and Bi (red) segregated along boundaries, and Cu (purple) uniformly distributed within the α-Al phase.

One of the main contributions of adding Bi to the Al–Sn system, from a tribological perspective, is the modification of the Sn-rich phase morphology, which changes from a reticular structure to a more globular one. This transformation is associated with improved wear behavior.^3,40^ Indeed, the most significant effect observed in the microstructure with the addition of Bi was the fragmentation of the Sn-rich secondary phase, as evidenced in the SEM micrographs shown in Figure 7b,c. This phenomenon occurs because Bi is immiscible with Al, which increases the interfacial tension between the Sn–Bi–rich liquid and the solid Al. As a result, wettability is reduced, promoting the formation of spherical droplets of the Bi-rich liquid instead of its growth along the Al dendrites. Concurrently, another portion of the Sn–Bi–rich phase distributes itself within the α-Al interdendritic regions, forming a continuous network structure characteristic of the monotectic reaction product.^35,40^

### 4.3 Wear Behavior

The worn volume (*mm* ^3^) as a function of sliding distance was transformed into wear coefficients according to the eq 2 and is shown in Figure 13. The wear coefficients for the Bi-modified alloys showed an irregular behavior, peaking at intermediate distances before sharply decreasing. Such a transition has been reported in previous studies and is often associated with the formation and stabilization of a solid lubricating layer on the contact surface.^41,42^ In contrast, the reference alloys show a more stable or steadily decreasing wear coefficient.

**Figure 13:**
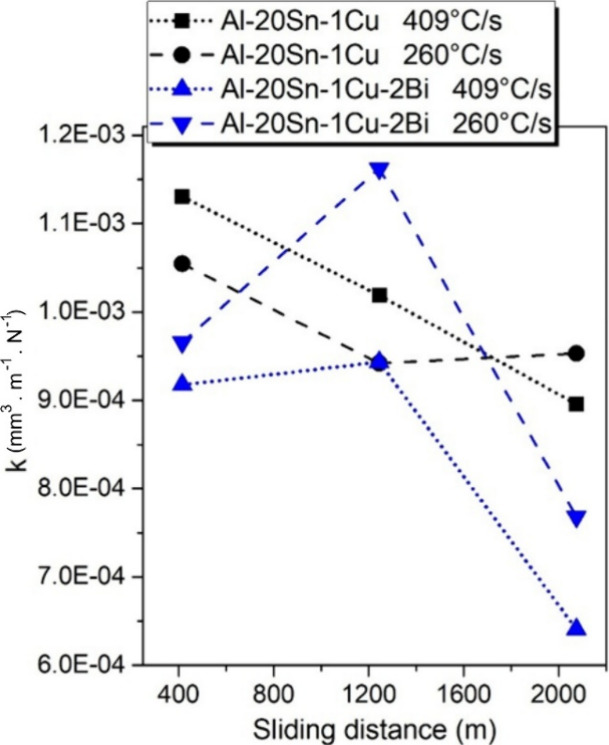
Wear coefficient (k) as a function of sliding distance for Al–20Sn–1Cu and Al–20Sn–1Cu–2Bi alloys solidified at 409 and 260 °C/s.

In self-lubricating alloys, it is well established that the type, distribution, and morphology of the soft phase are key factors governing wear behavior.^22,43^ In the present study, alloys with more refined microstructures consistently exhibited lower wear coefficients over longer sliding distances. This trend contrasts with reports on Al–Sn alloys solidified at intermediate cooling rates (<40 °C/s), where coarser and more segregated Sn-rich regions tend to promote higher wear.^25,42,44^ However, as suggested by Oliveira et al.,^36^ microstructural refinement enhances the spatial uniformity of the soft phase, and cellular or near-cellular morphologies may lead to improved tribological performance due to their more homogeneous phase arrangement. Thus, it is plausible that the increasingly refined structures observed here, approaching a cellular-like distribution of the soft phase, contributed directly to the progressive improvement in wear resistance over time.

Although microhardness is not typically the primary parameter controlling the wear coefficient in self-lubricating alloys,^42,44,45^ the present results show that the samples displaying higher hardness values also achieved superior wear resistance. This behavior is particularly pronounced in the Bi-modified alloy solidified at the higher cooling rate. In this condition, the refined microstructure combined with the presence of Bi leads to a steady decrease in the dimensional wear coefficient (k) with increasing sliding distance, ultimately reaching the lowest value among all tested conditions after 2000 m. Such evolution suggests the progressive stabilization of a lubricating film and a transition to a milder wear regime.

Two factors likely contribute to this improvement: (i) the significant increase in the hardness of the interdendritic region promoted by Bi addition,^1^ and (ii) the modification of the soft-phase morphology, which shifts from a fully reticular Sn network to a mixed reticular–globular arrangement with smaller and more uniformly distributed Sn–Bi regions.^40^ Together, these features enhance the mechanical stability of the surface during sliding while maintaining adequate solid lubrication, resulting in reduced wear severity. A similar tribological benefit associated with Bi addition has been reported by Dong et al.,^3^ who observed a reduction in the friction coefficient for an Al–20Sn alloy containing 2 wt % Bi and attributed this improvement to the presence of independently dispersed, granular Bi-rich soft phases.

SEM analysis of the worn surfaces revealed characteristics of adhesive wear, such as material pull-out and irregular craters, both seen in Figure 14. EDS analysis of the wear track (Figure 15) confirmed the adhesive mechanism, showing the presence of Fe and Cr, which indicates material transfer from the AISI 52100 steel ball to the sample surface. Evidence of oxidation within the wear track was also observed. However, there seems to be no significant difference in wear mechanism between the studied alloys in the present work.

**Figure 14:**
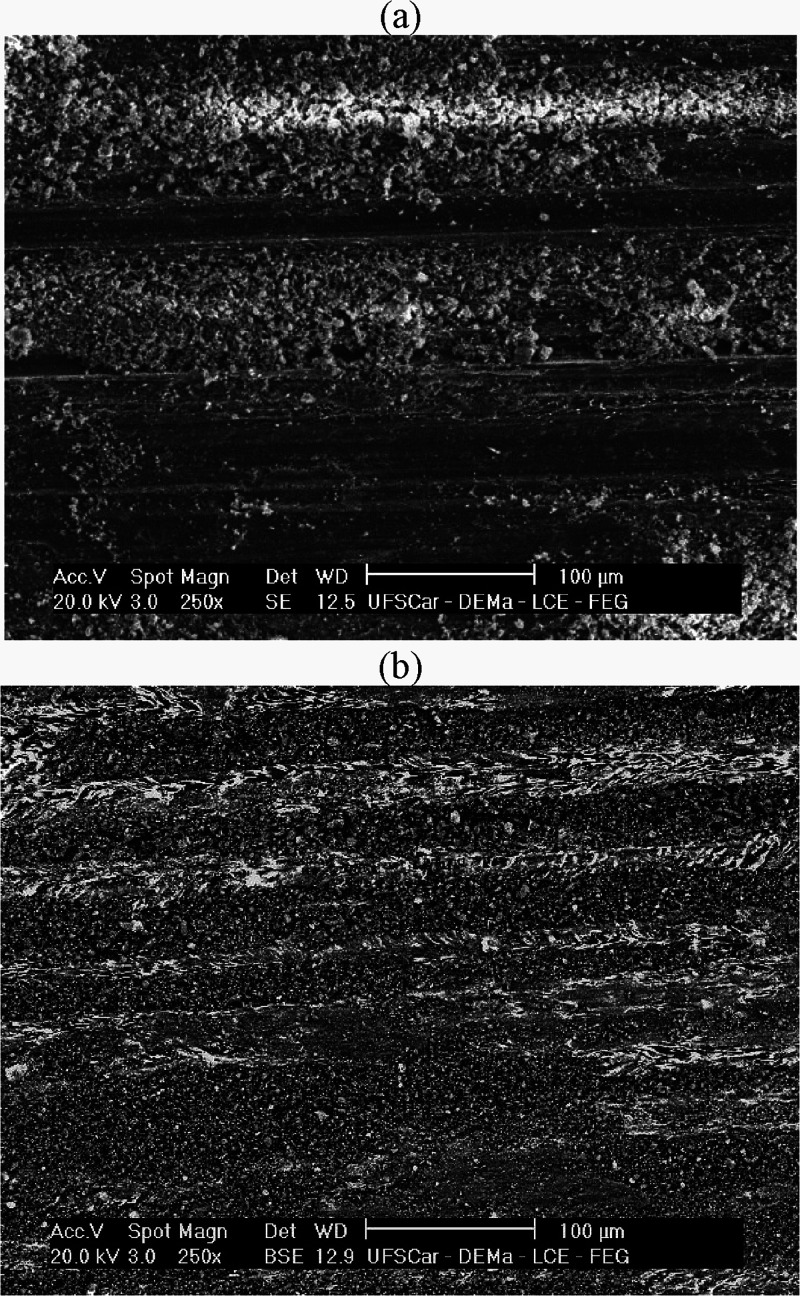
SEM micrographs of worn surfaces of (a) Al–20Sn–1Cu and (b) Al–20Sn–1Cu–2Bi alloys.

**Figure 15:**
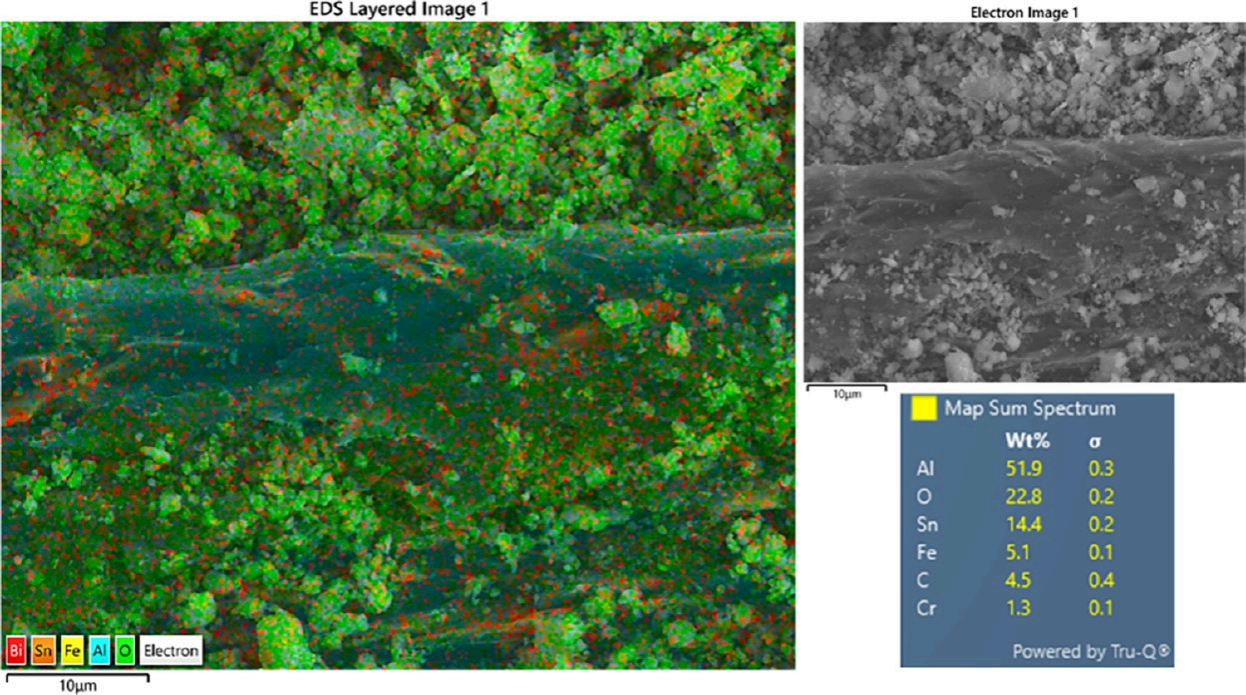
EDS dot map images of the worn surface of the Al–20Sn–1Cu–2Bi alloy.

## 5 Conclusions

Although coarse microstructures are traditionally considered the most favorable for self-lubricating Al–Sn alloys, the present results indicate that Bi addition becomes essential when the alloy is produced under rapid solidification conditions. In this scenario, Bi significantly decreases the wear coefficient along the sliding distance, promoting a transition to a milder wear regime. This improvement may be attributed both to the modification of the Sn-rich phase morphology, which becomes partially globular as well as to the overall increase in hardness induced by Bi addition. Therefore, for alloys solidified at high cooling rates, the incorporation of Bi emerges as an effective strategy to restore or even enhance the tribological performance of the Al–Sn alloys.
